# How to go beyond the body: an introduction

**DOI:** 10.3389/fpsyg.2015.00660

**Published:** 2015-05-21

**Authors:** Guy Dove

**Affiliations:** Department of Philosophy, University of LouisvilleLouisville, KY, USA

**Keywords:** embodied cognition, grounded cognition, extended cognition, perception, action, concepts

Embodied cognition represents one of most important theoretical developments in contemporary cognitive science. Many cognitive processes appear to be influenced by body morphology, emotions, and sensorimotor systems. This perspective is supported by an ever increasing collection of empirical studies that fall into two broad classes: one consisting of experiments that implicate action, emotion, and perception systems in seemingly abstract cognitive tasks and the other consisting of experiments that demonstrate the contribution of bodily interaction with the external environment to the performance of such tasks.

Now that embodied cognition is fairly well established, the time seems right for assessing its further promise and potential limitations. This research topic aimed to create an interdisciplinary forum for discussing where we go from here. Given that we have good reason to think that the body influences cognition in surprisingly robust ways, the central question is no longer whether or not some cognitive processes are embodied. Other questions have come to the forefront. To what extent are cognitive processes embodied? Are there disembodied processes? Among those that are embodied, how are they embodied? Is there more than one kind of embodiment? Is embodiment a matter of degree?

## Extending the research program

Many of the contributions to this research topic involve experiments that extend the empirical reach of embodied cognition. For instance, Soliman et al. (2013) ambitiously propose that sensorimotor mechanisms can unify explanations at cognitive, social, and cultural levels. They carried out two experiments investigating whether anticipated motor effort can be used to understand cultural differences. Building on earlier work by Proffitt and colleagues implicating an effect of perceived motor effort on visual distance perception (for a review see Proffitt and Linkenauger, 2013), they investigate a cultural motor-effort hypothesis in which relative degree of experience with out-group members can lead to differences in perceived distance. In a commentary, Wilson (2013) suggests that this effect conflicts with the task-relatedness of the effects found by Proffitt and colleagues. Soliman and Glenberg (2014) respond by clarifying how they link their cultural-motor effort hypothesis to the earlier work. Ultimately, further research is needed to settle these issues.

Much of the extant research on concepts within an embodied framework focuses on the binary question of whether or not they are embodied as a general rule. Recently, researchers have come to realize that embodiment might be context-dependent and come in degrees (e.g., Watson and Chatterjee, 2011; Pulvermüller and Garagnani, 2014; Zwaan, 2014). With this potential flexibility in mind, Watson et al. (2014) examined the sensorimotor specificity of action concepts elicited by different exemplars and representational formats. They found that actions appear to be represented at different levels of specifity by visual and motor systems and that the relative recruitment of some sensorimotor brain regions may depend on the format of the stimuli.

Abstract concepts remain a serious challenge for embodied cognition (Dove, 2015). A couple of the contributions address aspects of this challenge. Troche et al. (2014) defend a multidimensional approach to abstract concepts. Rather than rely on an intuitive notion of abstractness, they investigated how the meanings of 400 concrete and abstract English nouns are distributed in a multidimensional space using hierarchical cluster analysis. Participants rated the nouns along 12 dimensions. Factor reduction yielded three latent factors that the authors characterize as affective association, perceptual salience, and magnitude. When the original words were plotted for these three factors, abstract and concrete words were associated with unique, but somewhat overlapping, topographies within this space. Borghi et al. (2014) analyze how Italian Sign Language (LIS, Lingua dei Segni Italiana) encodes abstract concepts. They argue that the LIS data support the view that abstract concepts are encoded in multiple ways. Some abstract concepts may rely more on metaphors while others may rely more on situations, emotions, or linguistic information.

Despite the clear affinity between constructivist views of cognitive development and embodied cognition, the precise role that embodiment may play in development remains an open question. Corbetta et al. (2014) provide evidence suggesting that the emergence of reaching is a fundamentally embodied process. Infants appear to first learn to make such movements through the haptic and proprioceptive feedback associated with self-produced movements. Vision then maps onto this motor experience and contributes to the emergence of prospective motor control.

Although it is not always acknowledged, the conceptual re-framing of cognition as an embodied activity has important implications with respect to methodology. Bahnmueller et al. (2014) contend that near infrared spectroscopy (NIRS) is better suited to investigating the role that motion plays in embodied cognition than the more commonly used functional magnetic resonance imaging (fMRI).

## New directions

Several of the contributions are theoretical in nature. These echo many of the themes present in the experimental contributions but also expand the scope of embodied cognition. Some propose stronger versions of the embodiment thesis and others outline new frameworks for integrating embodied cognition with other disciplines.

Pouw et al. (2014) consider embodied theories of the cognitive function of gestures. As they see it, standard embodied accounts are too internalistic because they treat gestures as the epiphenomenal outputs of the sensorimotor processes involved in cognition. Pouw et al. argue that it would be more perspicuous to view gestures in terms of embedded/extended cognition (Kirsh, 1995; Clark, 2013; Wheeler, 2013) and treat them as external tools that can replace or support internal cognitive processes. In a related vein, Landy et al. (2014) defend an embodied account of symbolic reasoning in which external mathematical symbols and formulae serve as targets for action and perception systems. This account, which they refer to as Perceptual Manipulations Theory (PMT), suggests that mathematical and logical reasoning often involves the sensorimotor systems engaged by physical notations. Perceptual processes exploiting the design features of physical notations underwrite significant aspects of symbolic reasoning. Landy et al. contend PMT is supported by the growing body of evidence demonstrating the manifold ways that sensorimotor processes can influence or interrupt the capacity for symbolic reasoning.

One of the insights behind embodied cognition is that cognitive science has been overly concerned with higher-level cognition. We should instead pay closer attention to lower-level phenomena and consider the cognitive behavior of animals and less complicated agents. When we do, the importance of the body becomes apparent in ways that can be obscured when we focus only on higher-level cognition. Such a bottom-up approach has an underappreciated consequence: it raises significant questions concerning the ontogenetic and phylogenetic emergence of higher-level capacities.

On the ontogenetic front, Wellsby and Pexman (2014) suggest that work needs to be done in order to integrate embodied cognition with the large body of extant research on the development of concepts and language processing in children. They outline several important issues that need to be addressed in order to carry out this research program. Using ideas from radical embodied cognition (Chemero, 2009), Cowley (2014) proposes that there is a symbiotic relationship between linguistic embodiment and external verbal constraints. He offers a distributed-ecological account of how language skills emerge through the dynamic coordination of movement with verbal patterns and social experience.

On the phylogenetic front, Stutz (2014) suggests that an embodied approach can help illuminate the emergence of central human phenotypes such as linguistic communication and symbolic representation. His embodied niche-construction (ENC) hypothesis holds that these are the result of the dynamic co-evolution of embodied forms of cognition and changing environmental interaction. More specifically, it maintains that the capacity to form recursive iconic narratives was an important evolutionary precursor to the emergence of both. Gapenne (2014) defends the hypothesis that proprioception plays a fundamental role in the co-constitution of the self and the world by a cognitive system. He explicitly maintains that the coupling of proprioception and action is an important development in the phylogenesis of even simple organisms.

## Conclusion

The aim of this research topic was to bring together experts from multiple disciplines to discuss the future of embodied cognition. The resulting contributions suggest that embodied cognition is a robust and dynamic research program—one that is focused on addressing recognized challenges, exploring new empirical ground, and expanding its theoretical reach. Taken as a whole, they demonstrate the ongoing fecundity of this approach. Questions certainly remain, but that itself might be a good sign.

### Conflict of interest statement

The author declares that the research was conducted in the absence of any commercial or financial relationships that could be construed as a potential conflict of interest.
